# Influencing Factors of the Continuous Usage Intention of Consumers of Online Food Delivery Platform Based on an Information System Success Model

**DOI:** 10.3389/fpsyg.2021.716796

**Published:** 2021-08-16

**Authors:** Junbin Wang, Xiangdong Shen, Xinbei Huang, Yuting Liu

**Affiliations:** ^1^Business School, Changshu Institute of Technology, Changshu, China; ^2^School of Management, Fudan University, Shanghai, China; ^3^Southern Jiangsu Economic and Social Development Research Center, Changshu Institute of Technology, Changshu, China

**Keywords:** online food delivery, continuous usage intention, e-commerce, IS success model, influencing factors

## Abstract

Online food delivery (OFD) has gradually become a development trend in the catering industry. However, the homogenization of the principal OFD platforms is becoming increasingly obvious, and there is an urgent need to explore the continuous usage intentions of consumers of the OFD platform. Unlike previous studies, this study employs DeLone and McLean's information system (IS) success model as the theoretical framework and constructs the influencing factors model of the continued usage intentions of users of the OFD platform from three aspects, namely, service, system, and information quality. Participants (*N* = 361) completed a questionnaire assessing their continuous usage intentions of the OFD platform, and the results were analyzed using a structural equation model (SEM). Service worker, platform quality, and content consistency have significant positive effects on the trust in the platform and satisfaction of consumers. Both the trust in the platform and satisfaction of consumers have a positive impact on their continuous usage intentions. We also explored the mediating effect of the trust in the platform and satisfaction of consumers. This study adds to the academic literature and practical implications for the continuous usage intention of consumers. This study discusses the influencing factors and formation mechanism of the continuous usage intention of consumers and proposes a model of the continuous usage intention of consumers of OFD platforms. Moreover, OFD platforms can make differentiation strategies more targeted and eliminate the dilemma of homogenization competition of OFD platforms.

## Introduction

An online food delivery (OFD) platform provides an interconnecting online marketplace for restaurants, deliverers, and consumers who want their food delivered to their doorstep. Specifically, OFD apps allow customers to browse food at their favorite restaurants easily and then to quickly obtain the restaurant food they want through a delivery person. According to a survey by Statista, revenue in the OFD industry is expected to grow 9.9% annually from 2019 to 2023, with a market size of US$53.786 billion^1^. Grubhub, Deliveroo, and Uber Eats are just a few examples of these OFD platforms, a business model that is rapidly catching around the world.

Despite the rapid boom in the OFD industry, the competition between platforms for customers is becoming more intense because fewer customers are loyal to a single OFD platform. For example, in the first quarter of 2019, 62% of the customers of Grubhub did not use other OFD platform services, while it fell to 44% 2 years later^2^. The main reason is due to the trend toward homogeneity among OFD platforms. In other words, there is almost no difference in the features of the same product on different platforms. Taking the OFD market in China as an example, as reported by iResearch, a well-known marketing survey company, the main problems the OFD platforms facing at the present stage are tight profit space, difficulty in controlling customer audits, unclear market positioning, and serious homogenized competition^3^. In addition to the homogenization of price among OFD platforms, there is also obvious homogenization in market positioning, customer quality, delivery rider service, and other aspects. To break this deadlock, it is necessary to take the continuous usage intention of users of the OFD platform as the starting point and to analyze the different motivations and strengths of consumers in using the OFD platform. This study aims to help OFD platforms optimize platform operation in a targeted way and stand out from the homogenized competitive market.

Intention to continue using mobile apps is an important theoretical issue, as previous research has shown that the continued usage of mobile apps of consumers is lower than the expectations of developers (Lee and Kim, 2019). To improve continuous use or mobile shopping behavior, hedonistic-related factors, entertainment satisfaction, and mobile app atmosphere are generally considered to be critical influencing factors (Lee and Kim, 2019). However, online shopping for food is different from shopping for other things online (Liu and Lin, 2020). For the OFD system, unlike ordinary mobile applications, it integrates different online and offline entities such as information systems (IS), restaurants or merchants, delivery persons, and customers; therefore, it is difficult to understand the continued usage intention of consumers only from the perspective of IS. From the perspective of consumer psychology, the behavior of users of the OFD platform is affected not only by the quality of the IS itself but also by the service quality of the platform sellers and delivery people. It is precisely the influence of these comprehensive factors that shape the behavior of users of the OFD platforms, which is different from traditional IS. We believe that, in OFD systems, it is essential to understand the factors that influence the willingness of consumers to continue using and how they operate. In fact, previous studies have revealed that only a small percentage of online customers return to the original platform to make a purchase (Gupta and Kim, 2007; Qureshi et al., 2009). This study adopts DeLone and McLean's IS success model (D&M model) as our theoretical framework (DeLone and McLean, 1992).

On the theoretical basis of DeLone and McLean (1992), several issues related to the continuous use of IS have been explored, often referred to as the model of IS success. It identified six categories that explain the success of IS, namely, system quality, information quality, usage, user satisfaction, personal impact, and organizational impact. The D&M model has been proven to be a relatively mature theoretical model and has been widely used to predict the behavior of individuals in a variety of environments (Hsu et al., 2014; Tam and Oliveira, 2016; Aldholay et al., 2018; Lee et al., 2020; Mustafa et al., 2020; Shim and Jo, 2020). Moreover, DeLone and McLean (2004) argue that the D&M model can measure the degree of success of e-commerce. On online shopping sites, the fundamental role of information technology in facilitating transactions and communicating information to decision-makers has not changed. It is, therefore, reasonable to expect that, in the OFD system, the quality perception and satisfaction with the platform will affect the continued purchase intention of customers. However, few studies have examined what may affect the intention to continue buying in an e-commerce environment from the perspective of the D&M model. In addition, the standard business model for any OFD system can consider the four main actors of the platform, namely, the platform, restaurant or merchant, deliverer, and customer. This means that the perceptions and behaviors of customers may be influenced by their dual roles as platform users and buyers (Kim et al., 2004). That is, the continuous usage intention of consumers will be affected not only by the characteristics of the platform but also by the perception of the seller (Verhagen et al., 2006). In particular, the service attitude and efficiency of the delivery person are also critical factors. In this sense, previous literature using the D&M model to examine the influence of IS or the characteristics of e-commerce sites is limited in explaining consumer behavior of the OFD platform. To fill this gap, this study adopts the D&M model to test the predictors of the intention of consumers to continue using from the perspective of three aspects, namely, platform, restaurant or merchant, and delivery person. More precisely, the application of the OFD platform incorporates three specific components, namely, platform quality, content consistency, and service workers. Each factor indicates one facet of the D&M model related to IS or platform, that is, system quality, information quality, and service quality, respectively.

In general, this study investigates the antecedents and mediating mechanisms of the willingness of consumers to continue using OFD platforms by integrating the perspective of the D&M model and the unique characteristics of OFD platforms. Specifically, this study aims to answer the research questions as follows: (1) *Do quality perception (i.e., system, service, and information quality) and platform satisfaction (platform satisfaction and seller satisfaction) affect the willingness of consumers to continue using it?* (2) *Does trust in the platform affect the satisfaction and the willingness of consumers to continue using it?* (3) *What are the prerequisites for trusting the platform?* The findings may help scholars and practitioners gain insight into how to reposition the platform to stimulate the continued adoption of OFD platforms.

## Theoretical Model and Hypothesis Development

### Theoretical Model

**Figure 1:**
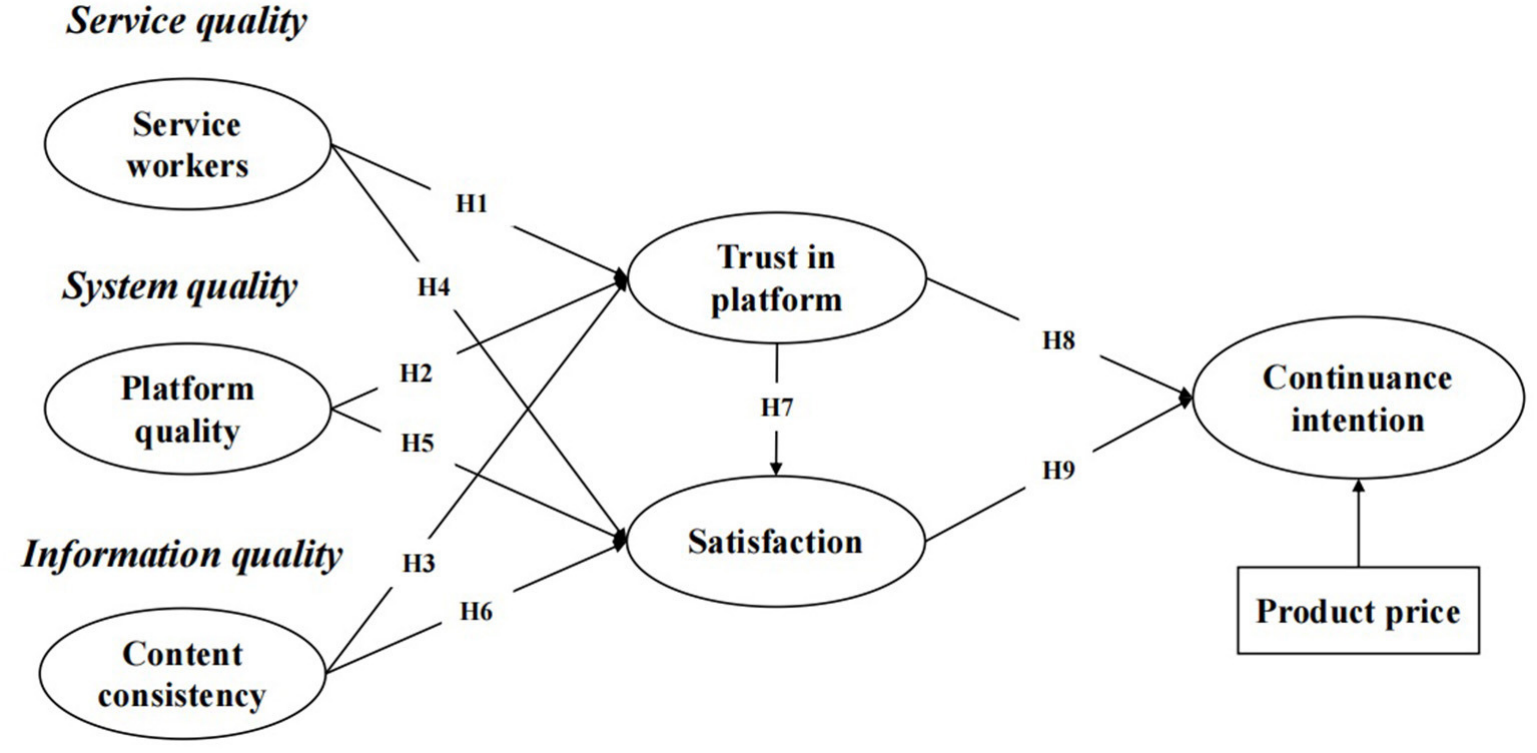
Research model.

Under the framework of the D&M model, the application of the OFD platform incorporates three specific components, namely, platform quality, content consistency, and service workers. Each factor indicates one facet of the D&M model related to IS or platform, that is, system quality, information quality, and service quality, respectively. This study examines the linkage between each facet of the platform and the continuance usage intention of consumers in OFD marketplaces and the mediating role of the trust in the platform and satisfaction of consumers. The conceptual model used in this study is presented in Figure 1.

### Hypothesis Development

#### Service Workers and Platform Trust

From the perspective of the D&M model, the counterpart to the service quality is the service workers. The critical role of service workers in providing quality service has been documented in the literature (Lee et al., 2019; Hong et al., 2020). In fact, the behavior of service workers greatly affects the perception of service of the customer. Furthermore, Nguyen (2006) found that service workers had a significant positive impact on corporate image. As an important part of the services provided by the OFD platform, service workers will undoubtedly have an important impact on the perceptions of consumers of platform quality. In an investigation of the impact of service quality, trust, and perceived value on customer loyalty, Rasheed and Abadi (2014) identified a significant positive correlation between service quality and trust. Moreover, Zehir et al. (2011) demonstrated that brand communication and service quality have a significant positive impact on brand trust. Therefore, service quality is conducive to promoting the trust of customers in the platform, and for OFD platforms, while service workers are closely related to service quality. This study, therefore, proposes the hypothesis as follows:

H1: Service workers have a significant positive impact on platform trust.

#### Platform Quality, Content Consistency, and Platform Trust

The other two dimensions of the D&M model, i.e., system quality and information quality, correspond to platform quality and content consistency, respectively. In online food ordering, since customers can only notice the limited information provided by the platforms, they cannot taste the food; thus, they cannot accurately judge the foods before they purchase them, which results in information uncertainty (Dong et al., 2021). To explore the impact of the perception of consumers of product quality, Wells et al. (2011) found that the quality of platforms will affect the perception of consumers of product quality; the higher the degree of information asymmetry, the greater the impact. Yoo and Donthu (2015) demonstrated that only high-quality shopping websites can attract consumers. It can be noted that a high-quality platform can enhance the purchase intention of consumers and gain their trust in the platform. In contrast, McKnight et al. (2017) examined the relationship between data quality and trust in business-to-business data exchange through the two-factor theory and the trust theory. Their results revealed that the information quality had a more significant positive impact on the trust of consumers. Similarly, if the foods received by customers are basically consistent with the content described by the platform, the OFD platform itself can provide a better image, which in turn enhances the trust of consumers. Therefore, this study proposes the hypotheses as follows:

H2: Platform quality has a significant positive impact on platform trust.
H3: Content consistency has a significant positive impact on platform trust.

#### Service Workers, Platform Quality, Content Consistency, and Satisfaction

With the rapid development of information technology, although OFD platforms can provide a variety of services, the diverse needs of customers cannot necessarily be met. Therefore, the type of quality the platform needs to provide to satisfy consumers is a problem. The antecedent investigation of the impact on consumer satisfaction has attracted the attention of researchers. In exploring mobile shopping applications on online-to-offline (O2O) platforms, Kim et al. (2021) found that good information quality and service quality had a positive impact on the perceptions of consumers of privacy, satisfaction, and loyalty. In a survey of satisfaction and the willingness of users to continue using chat robots, Ashfaqa et al. (2020) also found that such quality had a positive impact on consumer satisfaction. In the discussion about the influence of platform quality factors on user loyalty and continuous usage intention, Kim (2021) found that the impact of user interface on customers was also significant. In an investigation on online banking and how to satisfy customers to maintain competitiveness, Li et al. (2021) determined that security and service quality were the essential factors affecting customer satisfaction.

Service workers are in direct contact with consumers, which has an important impact on service quality. Platform quality plays a key role in the operation of the O2O system. When consumers order on the OFD platform, they mainly confirm through online information. If the information is inconsistent, it will cause consumer dissatisfaction. Although the impact of different fields on user satisfaction is different, according to the D&M model combined with the characteristics of OFD platforms, the antecedents of customer satisfaction can be assigned to service workers, platform quality, and content consistency (Ashfaqa et al., 2020; Kim et al., 2021). Under the mode of online and offline integration, consumers need to place orders online on the platform, which requires that the platform provides a fully functional and concise interface to facilitate users to operate on the platform. If the interface is too complicated, the effect of consumers will be greatly discounted. Moreover, customers also need to have direct contact with service workers offline. Communicating with a service worker with a good attitude will also make the shopping experience of the customer more pleasant. More importantly, whether the food is delivered on time and whether it is consistent with the description on the platform will directly affect consumer satisfaction. If the information provided by the platform is too different from what consumers really notice, they will have a great sense of gap, which is likely to lead to dissatisfaction. Therefore, the OFD platform needs to provide high-quality service workers, platform quality, and consistent content to improve consumer satisfaction. Thus, this study proposes the hypotheses as follows:

H4: Service workers have a significant positive impact on platform satisfaction.
H5: Platform quality has a significant positive impact on platform satisfaction.
H6: Content consistency has a significant positive impact on platform satisfaction.

#### Platform Trust, Satisfaction, and Continuance Intention

Derived from social psychology, trust has been conceptualized as the belief that the other person will act in accordance with appropriate behaviors such as generosity, integrity, and competence (Zhou, 2011). Zhang (2020) demonstrated that community trust has a significant positive effect on life satisfaction. Li et al. (2019) determined that the perceived usefulness of WeChat and the intensity of use had a mediating effect on the positive impact of institutional trust and life satisfaction, respectively. From trust to satisfaction is also a selective way to explain the trust mechanism in the consumer–seller relationship (Jung et al., 2020; Jeon et al., 2021). Chen and Chou (2012) and Javed and Wu (2020) argued that the greater the trust of the consumer, the greater their satisfaction with a transaction; in turn, their intentions to purchase or repurchase online will be enhanced. Similarly, from the perspective of OFD platforms, the more the customers trust the platform, the more satisfied they will be with it.

Many scholars have been concerned about the impact of the trust and satisfaction of customers on the willingness to continue using the platform. Kim et al. (2011) found that the continuance intention of consumers was realized by adjusting satisfaction and trust through three quality indicators, namely, service, system, and information quality. From the perspective of OFD platforms, trust and satisfaction with the platform have a direct impact on the continuous purchase intention of customers. Whether customers can trust and be satisfied with the platform becomes particularly important. Li and Fang (2019) explored the continuous usage intention of antecedents for brand application and found that brand attachment and satisfaction had a positive impact. In an antecedent survey of trust and continuous usage intention of mobile payment platforms, Shao et al. (2019) also identified a positive correlation between customer trust and continuous use. Only by gaining the trust of customers and by improving customer satisfaction, the OFD platforms can retain customers and improve their willingness for continuous usage. Based on the earlier analysis, this study formulates the hypotheses as follows:

H7: Platform trust has a significant positive impact on platform satisfaction.
H8: Platform trust has a significant positive impact on continuance intention.
H9: Satisfaction has a significant positive impact on continuance intention.

## Research Methodology

### Instrument

To ensure the causal relationship between antecedents and satisfaction, a pretest was conducted with participants drawn from the population for the main study. A total of 120 students (48% female) participated in return for course credit in a regular classroom setting. The results revealed that the trust in the platform and satisfaction of consumers with the merchant influence the continued usage of the platform app. To ensure content validity, the items used to measure the constructs were adapted from the existing literature and modified to fit the study context. The measurement items for service workers, platform quality, and information quality were adapted from the study by Shin et al. (2013). Trust in the platform was measured using four items adapted from the study by Pennington et al. (2003). Platform satisfaction was measured using four items adapted from the study by Chiu et al. (2012). Finally, continuance intention was measured by adapting three items from the study by Khalifa and Liu (2007).

As the original items were in English, we conducted a back translation to ensure translation validity. First, a researcher—whose native language is Chinese—translated the source items from English to Chinese. Then, another researcher independently translated these items back into English. Subsequently, the two researchers compared the two English versions and jointly revised the first Chinese version of the items. Based on their feedback, minor modifications were made to improve the comprehensiveness and user-friendliness of the measurement items. A pretest of the survey instrument was conducted to conceptually validate the instrument. The final survey questionnaire is presented in Appendix A. All items were measured on a 5-point Likert scale, ranging from 1 (not agree at all) to 5 (absolutely agree).

### Data Collection

The subjects for the study were the customers of the Meituan platform in China, who were members of the Meituan site or app of the firm. Meituan was selected for the reasons as follows: Meituan was launched in November 2013. With its market development experience and capital support, it has now covered major cities across the country and has become a giant in the OFD market. This study published a questionnaire on Sojump, which is the largest online survey platform in China, and invited users with the Meituan takeaway purchase experience to participate. The subjects were asked to answer questions based on their purchase experiences.

The data collection was conducted on March 2021. Users with Meituan OFD purchase experience were invited to participate. Participants were informed that their participation would assist in improving the OFD platform quality and that they would obtain a better customer experience as a result. The demographic characteristics of the final sample are summarized in Table 1. A total of 400 respondents were surveyed over a 4-week period. Finally, 361 responses were used for subsequent analyses after 39 incomplete and invalid responses were excluded. The participants were relatively balanced in gender distribution, and the majority (79.2%) were between 16 and 35 years of age. Furthermore, 73.1% of the respondents had an undergraduate or higher education, and 60.6% were students.

**Table 1:** Demographics of the survey respondents (*N* = 361).

**Demographics**	**Category**	**Frequency**	**%**
Gender	Male	153	46.8
	Female	174	53.2
Age	≤16	5	1.5
	16–25	115	35.2
	26–35	144	44.0
	36–45	44	13.5
	46–55	6	1.8
	≥56	13	4.0
Education	High school or below	44	13.5
	College student	239	73.1
	Graduate school or above	44	13.4
Occupation	Student	198	60.6
	Enterprise staff	48	14.7
	Public officials	63	19.3
	Unemployed	12	3.7
	Others	6	1.8

## Data Analysis and Results

### Reliability and Validity

Construct reliability and validity were further examined through the confirmatory factor analysis (CFA). As shown in Table 2, the Cronbach's α and composite reliability (CR) values for each construct ranged from 0.840 to 0.939, both of which were above the suggested threshold of 0.7 (Straub et al., 2004) and exhibited a satisfactory level of reliability. For construct validity, both convergent and discriminant validity were examined. The convergent validity was confirmed by examining the average variance extracted (AVE) and indicator loadings. As shown in Table 2, all the AVE-values were higher than the recommended level of 0.5 (Fornell and Larcker, 1981). The standard loadings of all items were above the desired threshold of 0.7 and significant at 0.001. This indicates good convergent validity (Chin et al., 1997).

**Table 2:** Results of confirmatory factor analysis.

**Construct**	**Indicator**	**Standard loading^a^**	**Cronbach's α**	**CR**	**AVE**
Service workers	SEW1	0.870	0.840	0.841	0.640
	SEW 2	0.737			
	SEW 3	0.787			
Platform quality	PLQ1	0.915	0.932	0.933	0.777
	PLQ 2	0.924			
	PLQ 3	0.793			
	PLQ 4	0.888			
Content consistency	COC1	0.824	0.846	0.847	0.649
	COC 2	0.760			
	COC 3	0.830			
Trust in platform	TRP1	0.823	0.915	0.915	0.729
	TRP 2	0.875			
	TRP 3	0.894			
	TRP 4	0.823			
Satisfaction	SAT1	0.849	0.936	0.936	0.785
	SAT 2	0.888			
	SAT 3	0.892			
	SAT 4	0.914			
Continuance intention	COI1	0.914	0.939	0.939	0.837
	COI 2	0.920			
	COI 3	0.911			

*χ^2^ = 1.541, CFI = 0.982, TLI = 0.978, GFI = 0.927, SRMR = 0.036, RMSEA = 0.041*.

a *All standard loadings were significant at p < 0.001*.

Discriminant validity was evaluated by comparing the square root of AVE and the correlation value. The discriminant validity was assessed by comparing the square root of AVE for each construct with the correlations between that construct and other constructs (Fornell and Larcker, 1981). As shown in Table 3, the square roots of the AVEs (i.e., diagonal elements) were larger than the inter-construct correlations depicted in the off-diagonal entries, thus suggesting the discriminant validity that was adequate.

**Table 3:** Results of discriminant validity testing.

	**Mean**	**S.D**.	**SEW**	**PLQ**	**COC**	**TRP**	**SAT**	**COI**
SEW	2.700	1.264	***0.800***					
PLQ	3.335	1.204	0.237	***0.881***				
COC	3.273	1.259	0.212	0.075	***0.806***			
TRP	2.483	1.238	0.460	0.204	0.264	***0.854***		
SAT	2.530	1.187	0.320	0.206	0.359	0.388	***0.886***	
COI	2.773	1.099	0.566	0.246	0.256	0.439	0.292	***0.915***

*SEW, service workers; PLQ, platform quality; COC, content consistency; TRP, trust in platform; SAT, platform satisfaction; COI, continuance intention. Diagonal bold italic entries are the square root of AVE; all others are correlations coefficients*.

As the data were self-reported from a single source, we performed two statistical analyses to assess the common method bias. First, we analyzed the data using the Harman's single factor test. We extracted six factors, and the variance explained by the most significant factor was only 32.984%. The results revealed that no single factor dominated the total variance, indicating a lack of common method bias. Second, we assessed the common method factor according to the steps suggested by Liang et al. (2007). The results demonstrated that the loadings of the principal variables were all significant at the *p* < 0.001 level, whereas none of the common method factor loadings were significant. These results further indicate that the common method bias is unlikely to be a concern in this study. In addition, a multicollinearity test was conducted to examine the correlations between independent variables. A variance inflation factor (VIF) value above 10 indicates a multicollinearity problem. As shown in Table 4, the VIF values for the variables were all below 10, indicating the absence of multicollinearity.

**Table 4:** Results of multicollinearity analysis.

**Model**	**Unstandardized coefficient**		**Standardized coefficient**	***t***	**Significance**	**Multicollinearity statistics**	
	**B**	**Standard error**	**β**			**Tolerance**	**VIF**
1(con.)^a^	0.619	0.212		2.919	0.004		
SEW	0.362	0.047	0.385	7.689	0.000	0.825	1.212
PLQ	0.106	0.044	0.112	2.376	0.018	0.934	1.070
COC	0.098	0.044	0.104	2.228	0.027	0.939	1.065
TRP	0.202	0.047	0.215	4.259	0.000	0.810	1.234
SAT	0.107	0.045	0.113	2.398	0.017	0.935	1.070

a *Dependent variable: continuance intention; SEW, service workers; PLQ, platform quality; COC, content consistency; TRP, trust in platform; SAT, platform satisfaction*.

### Hypothesis Testing

**Figure 2:**
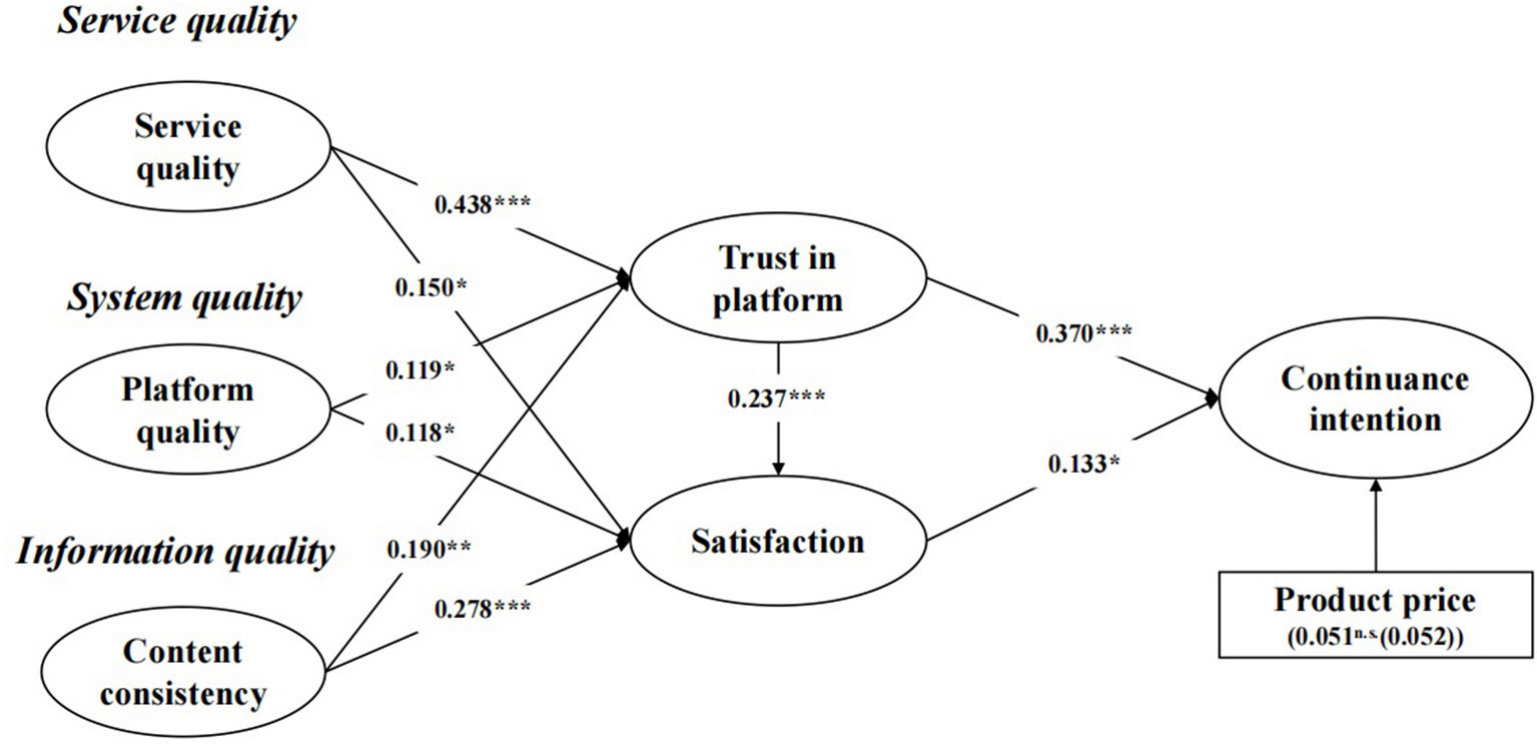
Results of the research model. *p < 0.05, **p < 0.01, and ***p < 0.001. n.s., not significance.

Figure 2 indicates that the nine hypothesized relationships are supported. Regarding service workers, both platform quality and content consistency had positive influences on trust in the platform. Additionally, service workers, platform quality, and content consistency had positive influences on platform satisfaction, thus supporting H1, H2, H3, H4, H5, and H6. Trust in the platform had a positive influence on platform satisfaction, thus supporting H7. In addition, trust and platform satisfaction had significant positive effects on continuance intention, thereby supporting H8 and H9. Among the three antecedents, service workers are found to have the greatest impact on trust in the platform (β = 0.438, *p* < 0.001), followed by content consistency (β = 0.190, *p* < 0.01), and the influence of platform quality is the least (β = 0.119, *p* < 0.05). Among the three antecedent factors of satisfaction, content consistency has the greatest impact on satisfaction (β = 0.278, *p* < 0.001), followed by service workers (β = 0.150, *p* < 0.05), and the influence of platform quality is the least (β = 0.118, *p* < 0.05) (Table 5).

**Table 5:** Hypotheses test.

**Hypothesis path**	**Path coefficient**	**SE**	***t*-Value**	***p*-Value**	**Results**
H1: Service workers → Trust in platform	0.438	0.066	6.621	^***^	Supported
H2: Platform quality → Trust in platform	0.119	0.056	2.127	0.033^*^	Supported
H3: Content consistency → Trust in platform	0.190	0.059	3.195	0.001^**^	Supported
H4: Service workers → Satisfaction	0.150	0.069	2.155	0.031^*^	Supported
H5: Platform quality → Satisfaction	0.118	0.057	2.087	0.037^*^	Supported
H6: Content consistency → Satisfaction	0.278	0.062	4.475	^***^	Supported
H7: Trust in platform → Satisfaction	0.237	0.066	3.572	^***^	Supported
H8: Trust in platform → Continuance intention	0.370	0.058	6.478	^***^	Supported
H9: Satisfaction → Continuance intention	0.133	0.054	2.445	0.014^*^	Supported

* *p < 0.05*,

** *p < 0.01*,

*** *p < 0.001*.

Then, to provide a more robust test of our results, control variables were included as direct antecedents of latent variables. According to the results of this study, the price of the product has no significant effect on the latent variables (β = 0.370, *p* > 0.5). In future research, we will take the price of the product as an independent variable to explore its impact on the continuance intention of customers (Figure 2).

Then, we examined trust in the platform and satisfaction mediation effects using the bootstrapping approach provided by Preacher and Hayes (2008). The use and test of the mediating effect is the main trend in management studies. In structural equation modeling (SEM), the conceptual model is a multiple mediator model, and there are two mediating variables (i.e., trust in the platform and platform satisfaction). As shown in Table 6, the indirect effect of trust in the platform and platform satisfaction on the relationship among characteristics of service workers, platform quality, content consistency, and continuance intention is significant with a 95% bootstrap CI, excluding zero. This finding suggests that trust in the platform and platform satisfaction mediate the effect of service workers, platform quality, and content consistency on the continuance intention of consumers.

**Table 6:** Results of mediating effect analysis.

**IV**	**M**	**DV**	**IV → M**	**IV → DV**	**M → DV**	**Indirect effect**	**CIs**	**Mediation**
SEW	TRP	COI	0.438^***^ (0.066)	0.423^***^ (0.062)	0.370^***^ (0.058)	0.192^**^ (0.039)	[0.120, 0.274]	Yes
PLQ			0.119^*^ (0.056)	0.106^*^ (0.049)		0.052^*^ (0.026)	[0.001, 0.105]	Yes
COC			0.190^**^ (0.059)	0.111^*^ (0.055)		0.083^**^ (0.029)	[0.034, 0.151]	Yes
SEW	SAT		0.150^*^ (0.069)	0.423^***^ (0.062)	0.133^*^ (0.054)	0.073^**^ (0.026)	[0.030, 0.127]	Yes
PLQ			0.118^*^ (0.057)	0.106^*^ (0.049)		0.042^*^ (0.019)	[0.009, 0.082]	Yes
COC			0.278^***^ (0.062)	0.111^*^ (0.055)		0.092^**^ (0.026)	[0.049, 0.155]	Yes
TRP	SAT		0.237^***^ (0.066)	0.370^***^ (0.058)	0.133^*^(0.054)	0.052^**^ (0.023)	[0.012, 0.105]	Yes

*95% Bootstrap CIs for the indirect effect*.

*IV, independent variable; M, mediator variable; DV, dependent variable; SEW, service workers; PLQ, platform quality; COC, content consistency; TRP, trust in platform; SAT, satisfaction; COI, continuance intention. IV → DV is significant (M not included in the model); IV → M is significant; M → DV is significant (or the meaningful reduction in effect) of the relationships between the initial IV and DV in the presence of mediator*.

*Significance at*

* *p < 0.05*,

** *p < 0.01, and*

*** *p < 0.001; SEs in brackets*.

## Discussions and Implications

### Discussion of Findings

This study yielded interesting findings. The results indicate that the quality of the OFD significantly influences the trust of consumers in the platform, their platform satisfaction, and their continuance intention in the future. First, regarding platform quality, service workers, platform quality, and content consistency were found to have significant impacts on the aforementioned factors. Moreover, service workers were found to have a stronger effect on trust in the platform (β = 0.438, *p* < 0.001), and content consistency has stronger effect on satisfaction (β = 0.278, *p* < 0.001). These findings are consistent with those of previous studies (Chiu et al., 2012; Tsao, 2013), indicating that platform quality significantly impacts trust in the platform and satisfaction.

Second, trust in the platform has a significant impact on satisfaction. Regarding the effects of trust and satisfaction on continuance intention, our results indicate that these factors can predict continuance intention and that trust in the platform (β = 0.370, *p* < 0.001) plays a greater role in determining continuance intention than platform satisfaction (β = 0.133, *p* < 0.05). This suggests that trust in a platform can induce a continuance intention of the consumer. Our findings extend those of previous studies (Eugene and Claes, 2000; Johnson et al., 2001), suggesting that satisfaction has a greater effect on continuance intention.

Finally, this study confirms the mediating effect of trust in the platform and satisfaction on platform quality and continuance intention. Our results confirm the mediating effect of satisfaction on trust in the platform and the continuance intention of consumers. The hypothesis is verified in line with the literature, highlighting the mediating effect of trust in the platform and satisfaction on platform quality and continuance intention (Izquierdo-Yusta et al., 2011; Legrand, 2013).

### Theoretical Contributions

Our research helps better understand the influencing factors of the emerging OFD platforms on the continuance usage intentions of consumers through the D&M IS success model. Using the D&M model, many studies have explored the influences of IS quality and satisfaction with IS on the continued usage of consumers in various contexts (Kulkarni et al., 2006; Wu and Wang, 2006; Lin, 2008; Teo et al., 2009). These studies documented that IS qualities have positive effects on the continued usage behavior and behavioral intention of consumers. However, research using the D&M model to examine the antecedents of continuance usage intention in the OFD platform is still scant. Similar to IS qualities that are considered as the key predictors of IS success, the qualities of the OFD platform also largely decide its success by maintaining the continuance usage intention of consumers.

Based on the D&M model, we identified three elements of the OFD platform quality, namely, platform quality, content consistency, and service workers. Each factor indicates one facet of IS quality, that is, system, information, and service quality, respectively. The findings indicate that service worker, platform quality, and content consistency have significant positive effects on the trust in the platform and satisfaction of consumers. These have a positive impact on their continuous usage intentions. We also explored the mediating effect of the trust in the platform and satisfaction of consumers. This study adds to the academic literature on the continuous usage intention of consumers regarding e-commerce platform applications. Our study also offers a new lens to evaluate when and how the OFD platforms can improve consumer loyalty, distinguishing our study from the existing IS or software management literature.

### Managerial Implications

This study offers useful managerial implications from two aspects. First, with the basic completion of the OFD market, user sharpening and perfect operations will become the focus of OFD platforms in the near future. How to retain and revitalize old users is a key to future competitiveness. Service workers, platform quality, and content consistency were found to have a significant positive impact on both the trust in the platform and platform satisfaction of users, which in turn affects the willingness of users to continue using it. Online food delivery platforms provide key factors affecting user stickiness to help OFD platforms improve their operations. In terms of specific operations, OFD platforms can enhance user insights, mine user needs, and strive to improve the quality of operations in terms of services, systems, and information through user interviews, browsing data analysis of the user, and text analysis of evaluation content of the user. According to the results of this study, to enhance the trust of customers in the platform, platform enterprises should pay attention to the service quality of service workers. In addition, to improve customer satisfaction with the platform, they should ensure the consistency of platform and product-information.

Second, compared with platform satisfaction, trust in the platform has a greater impact on the continuance intention of users, and it also has a significant positive impact on platform satisfaction. Therefore, trust in the platform should be another key point to which the OFD platform should continue to pay attention to and improve. Trust in the platform and satisfaction can be improved in the following ways: (1) The app is designed based on users rather than products or categories. It can be sorted according to distance, price, praise, sales volume, and so on, which is convenient for users to search and select. (2) User evaluation and rewards for printing pictures are introduced to improve the quality of information and update it in time for the reference of users. (3) Based on user insights, it provides functions such as merchant comparison, reminding orders, canceling orders, and refunding to improve platform service capabilities.

### Limitations and Future Research

Based on the IS success model, this study constructs a continuance intention model of the OFD platform and tests it by collecting user data from the Meituan platform. The results indicate that the constructed model is highly competent in explaining the problem. However, this research still has the following limitations. First, the Meituan platform is selected as the representative platform, and other OFD platforms may have certain differences. Second, this study does not consider the impact of price concessions on the continuance intentions of users of the OFD platform. In future research, user habits, price discounts, and so on can be considered as moderating variables to compare different OFD platforms.

## Data Availability Statement

The raw data supporting the conclusions of this article will be made available by the authors, without undue reservation.

## Ethics Statement

The studies involving human participants were reviewed and approved by the School of Business, Changshu Institute of Technology. Written informed consent for participation was not required for this study in accordance with the national legislation and the institutional requirements.

## Author Contributions

JW designed the study and drafted the initial manuscript. XS and XH collected the data, performed the statistical analysis, and drafted the initial manuscript. YL contributed to the revised manuscript. All authors discussed the results and contributed to the final manuscript.

## Conflict of Interest

The authors declare that the research was conducted in the absence of any commercial or financial relationships that could be construed as a potential conflict of interest.

## Publisher's Note

All claims expressed in this article are solely those of the authors and do not necessarily represent those of their affiliated organizations, or those of the publisher, the editors and the reviewers. Any product that may be evaluated in this article, or claim that may be made by its manufacturer, is not guaranteed or endorsed by the publisher.

## Footnotes

^1^Available at https://www.fatbit.com/fab/depth-feature-analysis-create-successful-online-food-delivery-platform/ (accessed May 23, 2021).

^2^Available at https://secondmeasure.com/datapoints/food-delivery-services-grubhub-uber-eats-doordash-postmates/ (accessed June 23, 2021).

^3^Available at http://report.iresearch.cn/report_pdf.aspx?id=2615 (accessed May 24, 2021, in Chinese).

## Appendix

**Appendix A:** Questionnaire items.

**Construct**	**Item**	**Source**
Service workers	SEQ1: I think the appearance of the food delivery person or rider is important	Shin et al., 2013
	SEQ2 I think the ability of a food delivery person or a rider is important	
	SEQ3: I think the professionalism of the food delivery person or rider is important	
Platform quality	SYQ1: I think the Meituan platform is of high quality	Shin et al., 2013
	SYQ2: I think the Meituan platform may be of high quality	
	SYQ3: I think the quality of the Meituan platform must be very good	
	SYQ4: I think the quality of the Meituan platform seems to be poor	
Content consistency	INQ1: I received the same response through online and offline channels	Shin et al., 2013
	INQ2: No matter online or offline, I can always receive the information sent to me by the merchant or platform	
	INQ3: Information is consistent between online and offline channels	
Trust in platform	PLT1: I think Meituan is trustworthy	Pennington et al., 2003
	PLT2: I think Meituan wants to be called a platform for keeping promises	
	PLT3: Meituan's behavior meets my expectations	
	PLT4: I believe that Meituan takes my best interests in mind	
Satisfaction	PLS1: Satisfaction after using Meituan is higher than other O2O platforms	Chiu et al., 2012
	PLS2: The overall satisfaction after using Meituan is very high	
	PLS3: Purchasing through Meituan is more economical than other O2O platforms	
	PLS4: I am very happy to be able to compare various products through Meituan	
Continuance intention	CUI1: I plan to continue using Meituan instead of discontinuing it	Khalifa and Liu, 2007
	CUI2: I want to continue to use Meituan instead of any other alternative platforms	
	CUI3: If possible, I want to stop using Meituan	
